# Human skin biomarkers relationship to response to treatment with tyrosine kinase inhibitors in advanced EGFR‐mutated lung adenocarcinoma

**DOI:** 10.1111/1759-7714.13657

**Published:** 2020-10-05

**Authors:** Anahí Castañeda‐Zárraga, Jerónimo Rafael Rodríguez‐Cid, Rodrigo Rafael Flores‐Mariñelarena, Héctor Trinidad‐Bibiano, José Fabián Martínez‐Herrera, Carla Paola Sánchez‐Ríos, Valeria Michelle Fernández‐Garibay, Jorge Arturo Alatorre‐Alexander, Luis Martínez‐Barrera, Patricio Javier Santillán‐Doherty, María Elisa Vega‐Memije

**Affiliations:** ^1^ Department of Dermatology Hospital General Dr. Manuel Gea Gónzalez Mexico City Mexico; ^2^ Department of Oncology Instituto Nacional de Enfermedades Respiratorias Ismael Cosío Villegas Mexico City Mexico; ^3^ Department of Internal Medicine Fundación Clínica Médica Sur Mexico City Mexico; ^4^ Department of Pathology Hospital General Dr. Manuel Gea Gónzalez Mexico City Mexico; ^5^ School of Medicine Monterrey Institute of Technology and Higher Education Mexico City Mexico; ^6^ Instituto Nacional de Enfermedades Respiratorias Ismael Cosío Villegas Mexico City Mexico

**Keywords:** Biomarkers, EGFR, NSCLC, skin biopsy

## Abstract

**Background:**

A relationship between the EGFR signaling pathway expression in skin and the use of targeted cancer therapies has been previously demonstrated. Consistent evidence to support the use of skin biopsies as a surrogate for therapeutic evaluation is needed. The purpose of this study was to establish the relationship between the expression of EGFR signaling pathway markers in skin samples from *EGFR*‐mutated metastatic lung adenocarcinoma patients and their response to tyrosine kinase inhibitors.

**Methods:**

This was a prospective single blind analysis of 35 skin biopsies from 31 patients with confirmed advanced *EGFR*‐mutated lung adenocarcinoma. Immunohistochemistry was performed: EGFR, p27, Ki67, STAT3 and MAPK, as well as H&E histopathological analysis, in order to determine their treatment response to tyrosine kinase inhibitors.

**Results:**

EGFR, Ki67, STAT3, stratum corneum thickness (number of layers and millimeters) from skin samples had a statistical correlation with an adequate treatment response (*P* = 0.025, 0.015, 0.017, 0.041, 0.039 respectively). EGFR, p27 and number of layers of the stratum corneum were related to a better median progression‐free survival (*P* = 0.025 and *P* = 0.030).

**Conclusions:**

The relationship between EGFR pathway inhibition in the skin and oncological outcomes obtained explains the parallel biological effects of tyrosine kinase inhibitors. We hope that our work incites future research to help validate and assess the use of these markers as potential prognostic and predictive factors.

## Graphical abstract

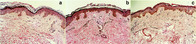

A total of 35 skin biopsies were obtained, of which 60% were female and 40% male, and the mean age of the participants was 60.6 ± 11.7 years. They were divided into three groups: pretreatment (12 patients: 34.3%); adequate response to treatment (12 patients: (34.3%); and no response to treatment (11 patients: 31.4%). We found that EGFR expression, STAT3, and the thickness of the stratum corneum (both in millimeters and in number of skin layers) had a statistically significant correlation with an adequate response to treatment with EGFR inhibitors in patients with stage IV lung adenocarcinoma. EGFR, p27 and the number of stratum corneum layers were related to better progression‐free survival. The relationship between EGFR inhibition in skin and in the tumor explains the parallel biological effect.

## Introduction

Lung cancer represents the number one cause of cancer‐related mortality worldwide, with an estimated two million new cases reported in 2018 alone, representing 11.6% of the total cancer incidence burden.^1^, ^2^ Reports of a five‐year survival rate of 5% in advanced stages calls for the use of targeted therapies and provides an explanation for the growing research in this field.^1^, ^2^, ^3^

Epidermal growth factor receptor (*EGFR*) mutation is one of the most common genetic disorders in non‐small cell lung adenocarcinoma, which is the most common lung cancer subtype worldwide, resulting in an ideal therapeutic target.^4^, ^5^, ^6^

EGFR has multiple functions in replication, survival and cellular homeostasis by signaling pathways such as phosphatidylinositol 3‐kinase (PI3K), Janus kinase (JAK) and RAS.^5^, ^7^, ^8^ EGFR expression is greatest at the basal and suprabasal layers of the epidermis and around the hair follicles, representing proliferating and more immature keratinocytes.^9^ The pharmacological inhibition of EGFR in lung cancer results in apoptosis and cellular growth arrest with systemic effects, not only in tumor cells, but also in keratinocytes.^10^ In skin samples taken from patients being treated with EGFR inhibitors, reduced expression of phosphorylated EGFR has been found in comparison with samples from healthy controls.^11^

A decrease in the proliferation of basal keratinocytes has been demonstrated by the reduced expression of markers such as Ki67 and an increase of p27 and other negative growth markers. Additionally, it has been observed that as EGFR inhibition increases, transcription factor, STAT3, levels also increase at the basal layer of the epidermis.^10^ Although STAT3 phosphorylation in human skin is not directly affected by EGFR activity, it may be an effect of the activation of alternating pathways triggered by cytokines and other growth factors.^10^

The inhibition of the aforementioned signaling pathways in patients treated with EGFR inhibitors produce documented histopathological skin changes, with the most frequent being: basket weave alteration of the stratum corneum (33%), spongiosis (79%–90%), perifollicular inflammation (80%–100%), infiltration by lymphocytes (100%), histiocytes (100%) and neutrophils (90%), sebaceous adenitis (70%), granuloma formation (60%), infiltrated dermis (59%), follicle inflammation (51%), perivascular infiltration (82%) vascular changes (100%) and vascular dilatation (82%).^12^, ^13^, ^14^, ^15^

The hypothesis is that skin sample studies from *EGFR*‐mutated lung adenocarcinoma patients allow us to establish a relationship between EGFR expression or its signaling pathway derivatives, and gefitinib treatment response, potentially acting as a tool to avoid tumor biopsy related risks.

Receiver operating characteristic (ROC) curve analysis was applied to set cutoff value parameters for EGFR, P27, Ki67, STAT3, and MAPK expression. Finally, clinicopathological and prognostic significance of biomarker's expression in skin tissue samples were analyzed.

## Methods

A prospective single blind study was performed at the Thoracic Oncology and Pathological Anatomy departments at the “Instituto Nacional de Enfermedades Respiratorias, Ismael Cosio Villegas” (INER), in Mexico City. The study design was approved by INERs Institutional Ethics Board in accordance with the Declaration of Helsinki, Fortaleza Brazil 2013 (approval document: C25‐18). Patients older than 18 years old with a histopathological diagnosis of *EGFR*‐mutated lung adenocarcinoma, treated with gefitinib and ECOG ≤2 were included. Exclusion criteria were: patients with cutaneous disease at the time of the study, concomitant treatment with systemic steroids (prednisone ≥10 mg/day or equivalent), previous treatment with TKIs, concomitant use with other antineoplastic treatments, patients undergoing anticoagulant treatment, impaired healing, or patients with immunological diseases, pregnancy, lactation, and nonevaluable treatment responses. A signed inform consent form was obtained from each patient included in the study. The immunohistochemical and financial related issues were absorbed by the investigation group from the study.

## Tissue samples

An expert dermatologist performed 35 skin biopsies from 31 patients with a confirmed diagnosis of stage IV *EGFR*‐mutated lung adenocarcinoma, taken by 4 mm punch in the scapular region, which was subsequently formalin‐fixed and paraffin‐embedded. Patient samples were classified into three groups: (i) Pretreatment; (ii) adequate treatment response (complete and partial response); and (iii) no treatment response (stable disease and progression) according to RECIST 1.1 criteria assessed by our group's radiologist with a computed tomography (CT) scan. One patient was included in three groups (pretreatment, adequate response to treatment and no response to treatment), and two patients were included in two groups (adequate response to treatment and no response to treatment); these biopsies were taken at different times during the patients’ evaluation according to RECIST 1.1 criteria.^16^ Skin biopsies of patients in groups 2 and 3, were taken at the consultation with the oncologist which took place 24 hours after the image study and at least three months after the start of treatment and at any time during the treatment.

### Histopathological analysis

**Figure 1:**
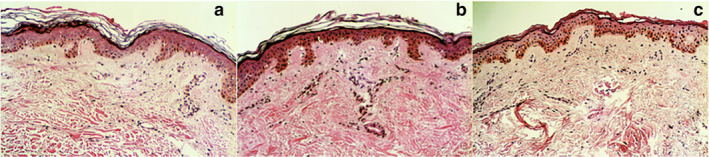
Histological findings from skin biopsies for each group. (**a**) Pretreatment. (**b**) Adequate response to treatment; note the changes in the configuration and fewer layers in the stratum corneum. (**c**) Patient with tumor progression; the stratum corneum does not appear to have a normal configuration.

Histopathological analysis with hematoxylin and eosin staining was performed to confirm stratum corneum configuration, stratum corneum thickness measured in micrometers and number of layers, parakeratosis, spongiosis and infiltrate (type, disposition and depth). The measurement mentioned above was taken from the top of the stratum corneum to the top of the granular cell layer at several sites (Fig 1).

### Immunohistochemistry

**Figure 2:**
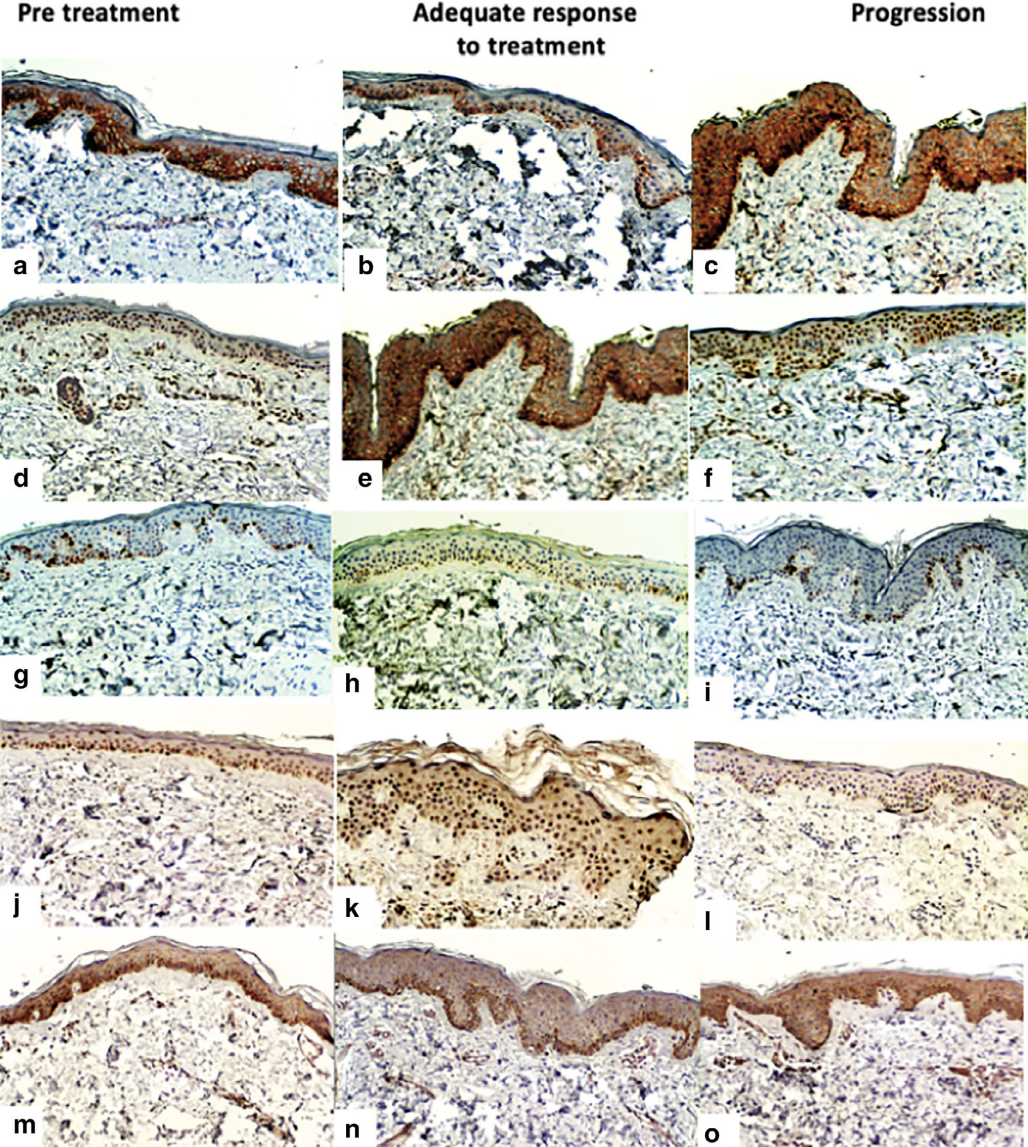
Immunohistochemistry of skin biopsies. (**a**, **b**, and **c**) EGFR immunohistochemistry staining. EGFR expression was diminished after treatment, especially in patients with an adequate response to treatment. (**d**, **e**, and **f**) p27 immunohistochemistry staining; patients with an adequate response showed increased expression in the epidermis. (**g**, **h**, and **i**) Ki67 immunohistochemistry staining; patients on treatment had a lower expression of Ki67, especially those with an adequate response to treatment. (**j**, **k**, and **l**) STAT3 immunohistochemistry staining; patients with an adequate response to treatment had a higher expression of STAT3. (**m**, **n**, and **o**) MAPK immunohistochemistry staining; patients with tumor progression showed an increased expression of MAPK.

Immunohistochemical analysis measured the percentage of expression of EGFR (3167, Biocare Medical), p27 (SX53G8, Dako), STAT3 (GTX118000, Genetex), MAPK (GTX50566, Genetex) and Ki67 (SP6, Biocare Medical). The number of cells in the basal layer of the epidermis were also counted (Fig 2).

### Immunohistopathology evaluation

The histopathological and immunohistochemical analysis performed on the biopsy samples was performed by our group's dermatopathologist, who was blinded to clinicopathological patient data.

### Statistical analysis

**Figure 3:**
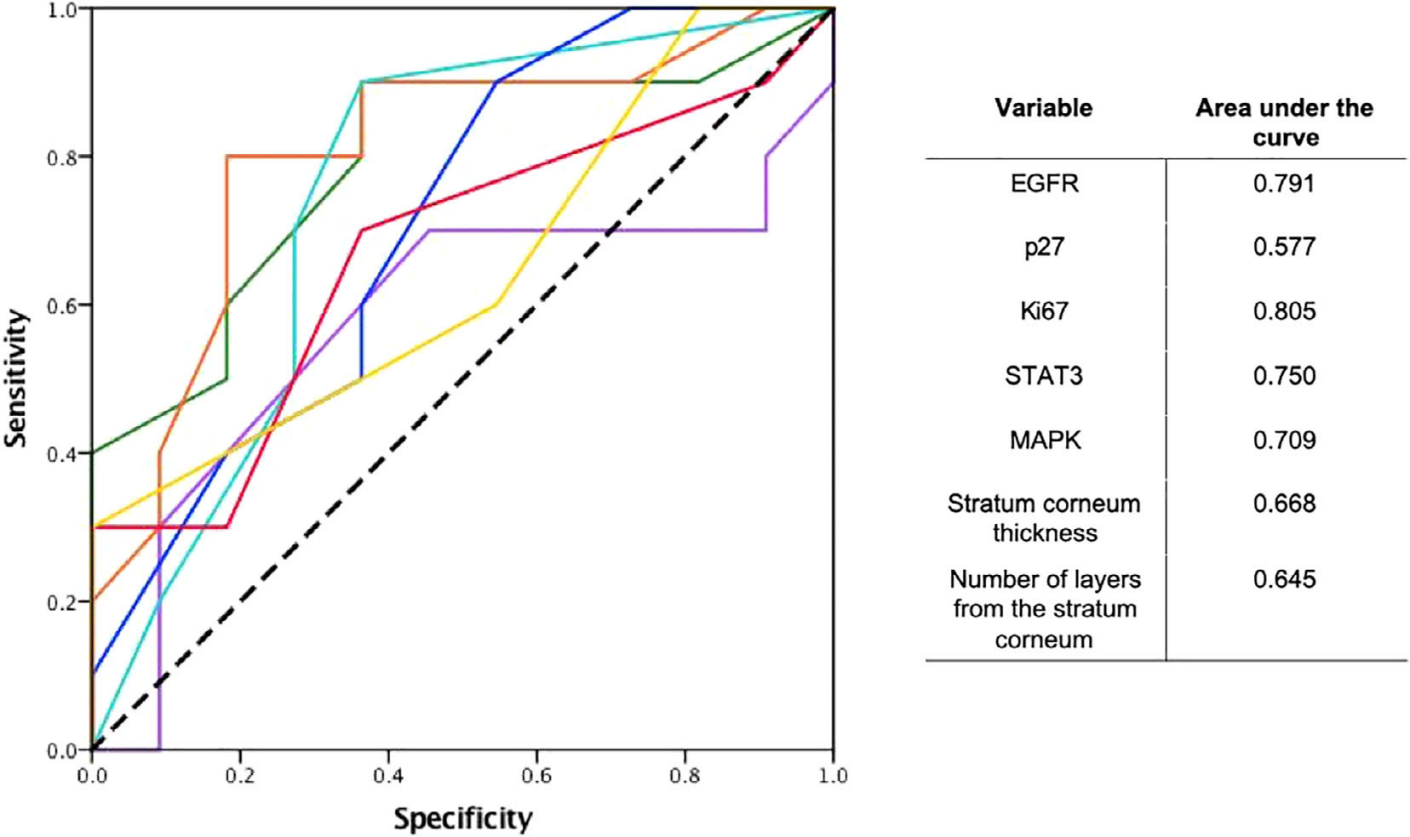
Receiver operating characteristic curve (ROC) analysis to determine the cutoff value for 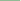, EGFR; 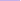, p27; 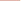, Ki67; 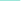, STAT3; 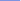, MAPK; 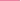, stratum corneum thickness; and 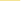, number of layers from the stratum corneum.

SPSS software version 24.0 (IBM software, Armonk, NY, USA) was used for statistical analysis. A ROC curve analysis was used to determine the cutoff value for each biomarker, using the shortest distance from the curve to the point with the maximum sensitivity and specificity (0.0, 1.0) dichotomizing the response to treatment as mentioned in the tissue samples section (adequate response or no response), to compare changes between the two groups (Fig 3).^17^ Correlation between immunohistochemistry and histopathological changes with response to treatment in patients treated with TKIs (adequate response and no response to treatment) was evaluated using the Pearson correlation coefficient. Univariable and multivariable survival analyses were performed using a logistic regression model, and survival curves were plotted using the Kaplan‐Meier method. The variables were expressed as the median values, as well as total values and percentages. The criterion for statistical significance was *P* < 0.05.

## Results

From the 35 biopsies obtained, 21 (60%) of the patients were women and 14 (40%) men; mean age of participants was 60.6 ± 11.7 years. From the three groups previously described in the methods section, 12 (34.3%) were in the pretreatment group, 12 (34.3%) had an adequate response to treatment and 11 (31.4%) were in the no response to treatment group. The mean duration of treatment was 8.6 months with a median progression‐free survival of nine months.

The ROC curve analysis defined the next cutoff values to consider a positive change between the pretreatment patients and post‐treatment groups: EGFR 72.5%, p27 45%, MAPK 45%, Ki67 61 keratinocytes, STAT3 0.5 keratinocytes, thickness of the stratum corneum 0.025 μM and 3.5 layers from the stratum corneum, these values represent a change from the basal state of 5.83%, 3.75%, 18.75%, 10 keratinocytes, 1.5 keratinocytes, 0.0083 μM and 0.6 layers, respectively. These cutoff points were pre‐established by using baseline means and performing a preanalytical ROC curve to set the change parameters.

The next biomarkers were significantly related to an adequate response to treatment by using a bivariate correlation test: EGFR (*P* = 0.025), Ki67 (*P* = 0.015), STAT3 (*P* = 0.017) stratum corneum thickness (*P* = 0.039) and number of layers in the stratum corneum (*P* = 0.041). A tendency to statistical significance was found with the use of MAPK (*P* = 0.059).

**Figure 4:**
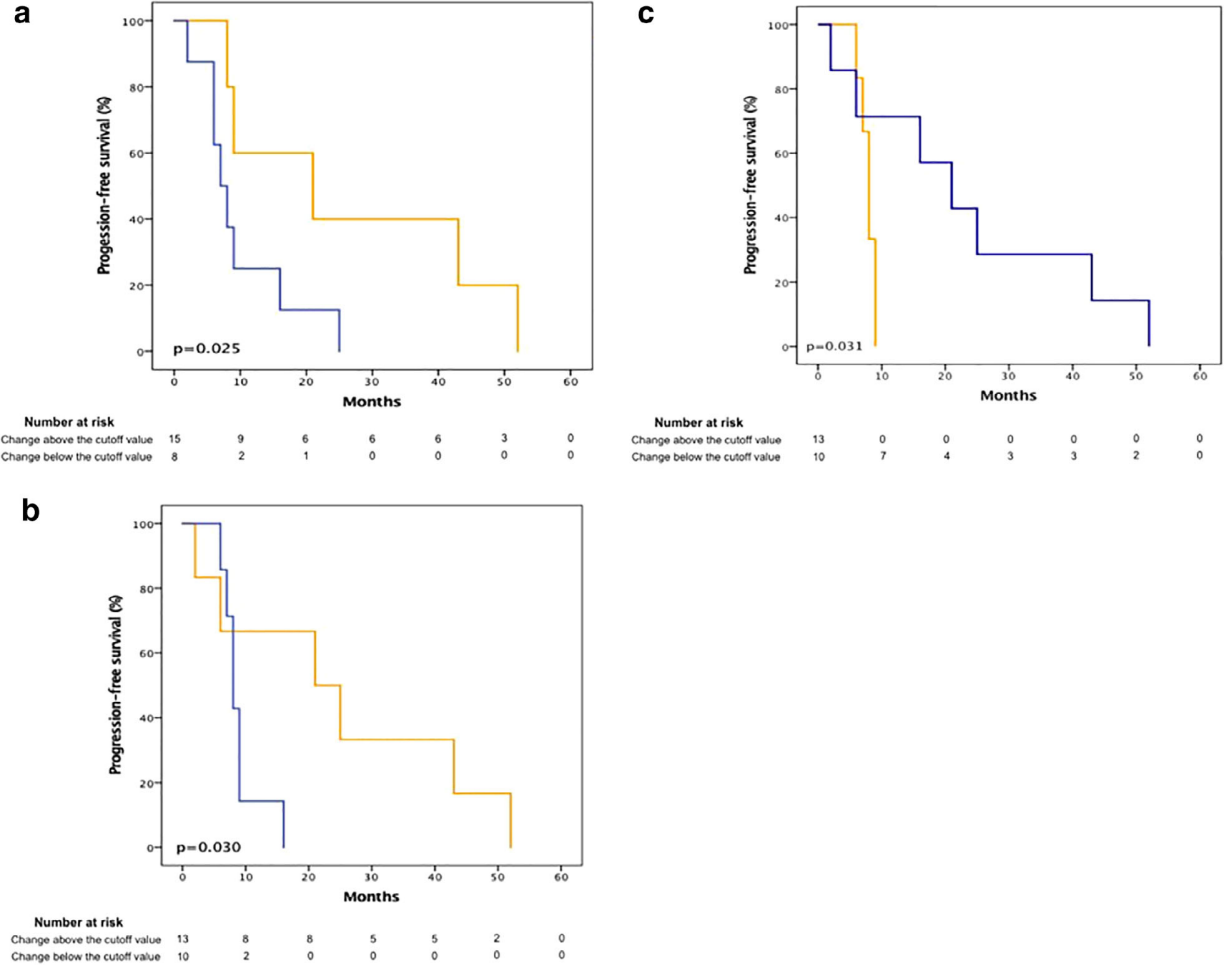
Progression‐free survival of patients treated with a tyrosine kinase inhibitor in months, compared the patients with an expression above the pre‐established cutoff values against those below. (**a**) EGFR 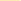, Change above the cutoff value; 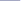, Change below the cutoff value. (**b**) number of layers in the stratum corneum 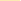, Change above the cutoff value; 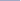, Change below the cutoff value. (**c**) p27 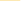, Change above the cutoff value; 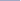, Change below the cutoff value.

Using the aforementioned post‐treatment cutoff values, a Kaplan Meier analysis for progression‐free survival was performed, dichotomizing and comparing those with a value above, against those with a value below. A better median progression‐free survival was obtained on those with a value above the EGFR pre‐established cutoff (21 months vs. seven months, 95% CI: 0–46 vs. 4.23–9.77, *P* = 0.025) and number of layers in the stratum corneum (21 months vs. eight months, 95% CI: 0–43.81 vs. 6.72–9.28, *P* = 0.030); however, for p27 a better median progression‐free survival was shown in those with a value below the cutoff mentioned previously (21 months vs. eight months, 95% CI: 8.17–33.83 vs. 6.87–9.13, *P* = 0.031) (Fig 4).

No statistical correlation was found in other analyzed biomarkers for treatment response or progression‐free survival.

## Discussion

In the present study, a relationship between response to treatment with EGFR inhibitors in patients with stage IV lung adenocarcinoma, and the expression of EGFR in skin as well as the number of layers in the stratum corneum was found. In patients treated with EGFR inhibitors, EGFR expression was diminished both in the tumor as well as skin samples, which demonstrates the parallel and simultaneous biological effects of these drugs, making it possible to determine the stage of blockade of EGFR with a skin biopsy analysis.^18^, ^19^, ^20^

EGFR inhibition occurs primarily at the start of treatment, and there is a tendency in patients with an adequate treatment response to show lower EGFR levels when compared with patients facing disease progression, or those in the pretreatment phase. Similarly, there is also a tendency in patients with progression to present with higher EGFR levels than patients with an adequate response to oncological treatment.

These findings are related to the systemic inhibition of EGFR, which has effects on the skin by reducing keratinocyte proliferation and migration to upper layers in the epidermis. In addition, this is also probably due to the fact that the EGFR pathway has not been effectively blocked in patients who present with disease progression, and they come close to the basal levels observed in pretreatment patients. Considering that these lung cancer patients receive a tyrosine kinase inhibitor aimed at their specific *EGFR* mutation, the expression of the mutated gene and its products is diminished both in malignant and normal cells, such as those found in skin. This serves to explain how patients with an adequate treatment response have a superior inhibition of the EGFR pathway (in skin and neoplastic cells) than those with disease progression or pretreatment. By facing disease progression, the tumor is able to gain treatment resistance and find genetic escape routes which allow tumor proliferation. To date, the amplification of EGFR, has not been identified as one of those escape routes, which is why we have not found differences in cutaneous EGFR expression at the time of disease progression.

Ki67 is a nuclear protein present in cell cycle growth phases (G1, S, G2, M) used as a proliferation marker.^15^ Treatment with tyrosine kinase inhibitors inhibits cellular proliferation and increases apoptosis,^21^, ^22^, ^23^ which is why the effective inhibition of EGFR present in patients with adequate treatment response is reflected in the lower Ki67 levels found in skin.^10^ In accordance with reports by Albanell *et al*.^18^ our study shows that epidermal cell proliferation decreases after exposure to tyrosine kinase inhibitors against EGFR. We found more Ki67 positive keratinocytes in pretreatment than post‐treatment patients. Patients with an adequate treatment response had lower Ki67 levels than pretreatment patients. Patients with adequate response also showed a tendency to express lower Ki67 levels than patients without response, and subsequently, patients without response showed lower Ki67 levels than pretreatment patients.

CDKN1B or p27 is a tumor suppressor protein that regulates cell cycle progression from G0 to S phase; induced by tyrosine kinase inhibitors to halt tumor cell proliferation.^10^, ^24^, ^25^ As a result, it would be expected for p27 expression to rise after treatment, especially in patients with an adequate treatment response.^24^ However, our study did not find statistically significant changes, although a tendency to express higher p27 levels was noted in patients with an adequate response when compared to those without response and in pretreatment patients. The increase of p27 expression appears to be related to an increase of median progression‐free survival. This is contrary to the findings reported by Busam *et al*.^26^ who found that p27 levels increased in patients treated with cetuximab at the eighth day of treatment; differences that could be explained by the time of biopsy collection or the treatment agent used in each study. Additionally, to date, cetuximab does not have any effect in patients with *EGFR* mutations.

In contrast to other studies, the expression of Ki67, STAT3 and MAPK in skin did not show a relationship with adequate treatment response with EGFR inhibitors related to progression‐free survival in patients with stage IV lung adenocarcinoma. However, this may be related to the skin biopsy collection times, since our skin biopsies were taken at different times and there may be escape routes for the keratinocytes to adapt to EGFR inhibition.^10^, ^18^, ^27^ In contrast to previous reports in the literature, we did not observe changes in the epidermis such as spongiosis or parakeratosis, and most of our biopsies presented only mild superficial perivascular lymphocytic infiltrates.^12^, ^13^, ^14^, ^15^, ^28^

We also found that the EGFR inhibition of the tumor was related to skin alterations, such as stratum corneum thickness. The systemic inhibition of EGFR has been previously reported to have effects on the skin by decreasing the proliferation, migration and survival of keratinocytes towards the upper layers of the epidermis.^7^, ^11^, ^25^, ^29^, ^30^ Albanell *et al*.^18^ reported that changes observed in the epidermis were related to treatment response, since patients with an adequate response to treatment are those who present with major changes at the stratum corneum (configuration and thickness), even when compared with patients without treatment response. The inhibition of EGFR in skin was related to a better median progression‐free survival, which explains the strong relationship between EGFR expression in skin and the tumor response.

Similarly, a multicentre, phase 2 study in which non‐small cell lung cancer (NSCLC) patients were given increasing doses of erlotinib, showed altered differentiation of epithelial structures in biopsies collected during treatment when compared with pretreatment biopsies.^31^ Some studies even suggest that the appearance of skin conditions such as epidermal growth factor inhibitor (EGFRI) toxicity‐related skin rash could potentially be a predictive marker for treatment efficacy, since they reflect drug concentration and activity.^19^, ^32^, ^33^, ^34^, ^35^

The potential limitations in this study are related to the relatively small number of patients who participated in our analysis. Additionally, since our research center is a highly specialized referral institution, generalizing these results to patient populations at other medical institutions need to be analyzed further. Finally, even when the overall genetic effect of EGFR blockade was observed, the gene expression itself was not evaluated.

In conclusion, the parallel biological effects of systemic EGFR pathway inhibition is observed in both tumor response and skin biopsies in patients with advanced lung adenocarcinoma. In this study, patients treated with tyrosine kinase inhibitors showed reduced EGFR and Ki67 expression, especially evident in those who presented with an adequate treatment response. Alterations in the configuration of the stratum corneum and decrease in its number of layers were also found to be related with treatment response and the start of treatment with tyrosine kinase inhibitors. No relationship between p27 and response to oncological treatment was found.

We found a relationship between EGFR, stratum corneum and number of layers in the stratum corneum, in patients with treatment response. We also found that there was a better progression‐free survival for patients with high expression EGFR, decreased number of layers in the stratum corneum and low p27 expression. The relationship between EGFR pathway inhibition in the skin and the oncological outcomes obtained, explains the parallel biological effects of these agents.

This study presents strong evidence that EGFR lowered expression in skin samples from stage IV lung adenocarcinoma patients treated with tyrosine kinase inhibitors could potentially be used as a surrogate to predict treatment response and avoid tumor biopsy‐related risks in this population. We hope that our work incites future research to help validate and assess the use of these markers as potential prognostic and predictive factors.

## Disclosure

Dr Jeronimo Rafael Rodríguez‐Cid has educational, investigational and advice relations with MSD, Bristol Myers, Roche, Takeda, Amgen, Abvie, Aztra Zeneca, Boehringer Ingelheim, Pfizer, Celgen, Novartis and Bayer; Dr Jorge Arturo Alatorre‐Alexander has educational, investigational and advice relations with MSD, Bristol Myers, Roche, Takeda, Aztra Zeneca, Boehringer Ingelheim and Pfizer. The other authors have no conflicts of interest to declare.
